# CaMK4 Promotes Acute Lung Injury Through NLRP3 Inflammasome Activation in Type II Alveolar Epithelial Cell

**DOI:** 10.3389/fimmu.2022.890710

**Published:** 2022-06-06

**Authors:** Tengyue Zhang, Mengyuan Li, Siyuan Zhao, Mianjing Zhou, Huai Liao, Haiyan Wu, Xinyue Mo, Hongxing Wang, Chaohuan Guo, Hui Zhang, Niansheng Yang, Yuefang Huang

**Affiliations:** ^1^ Department of Pediatrics, First Affiliated Hospital, Sun Yat-sen University, Guangzhou, China; ^2^ Department of Rheumatology, First Affiliated Hospital, Sun Yat-sen University, Guangzhou, China; ^3^ Department of Pulmonary and Critical Care Medicine, First Affiliated Hospital, Sun Yat-sen University, Guangzhou, China; ^4^ Institute of Precision Medicine, First Affiliated Hospital, Sun Yat-sen University, Guangzhou, China

**Keywords:** acute lung injury, CaMK4, type II alveolar epithelial cell, NLRP3 inflammasome, IL-1β

## Abstract

**Background:**

Type II alveolar epithelial cell (AEC II), in addition to its roles in maintaining lung homeostasis, takes an active role in inflammatory response during acute lung injury (ALI). Ca^2+^/calmodulin-dependent protein kinase IV (CaMK4) activated by Ca^2+^/calmodulin signaling, has been implicated in immune responses. This study was to investigate the roles of CaMK4 in the development of ALI and the underlying mechanisms.

**Methods:**

CaMK4 inhibitor KN-93 was used to investigate the effects of CaMK4 on NLRP3 inflammasome activation. The effects of KN-93 on disease development of lipopolysaccharide (LPS)-induced ALI were also evaluated. The role of CaMK4 on NLRP3 inflammasome activation was explored in human AEC II cell line A549 using KN-93 or CaMK4 siRNA. NLRP3 inflammasome activation was measured by histology immunofluorescence and Western blot. IL-1β and IL-18 were measured by ELISA.

**Results:**

Phosphorylation of CaMK4 and the expression of NLRP3 and Caspase-1 p20 were increased in the lungs of LPS-induced ALI mice, which was suppressed by KN-93 as measured by Western blot. Further, the activation of NLRP3 inflammasome was detected in AEC II from patients with acute respiratory distress syndrome (ARDS) and LPS-induced ALI mice. *In vitro*, inhibition or silencing CaMK4 in AEC II significantly inhibited NLRP3 inflammasome activation, resulting in reduced IL-1β production. The inhibition of NLRP3 inflammasome and decreased IL-1β/IL-18 production by KN-93 led to reduced inflammatory infiltration and ameliorated lung injury in LPS-induced ALI mice.

**Conclusion:**

CaMK4 controls the activation of NLRP3 inflammasome in AEC II during LPS-induced ALI. CaMK4 inhibition could be a novel therapeutic approach for the treatment of ALI.

## Introduction

Acute lung injury (ALI) is a critical syndrome predisposed to acute respiratory distress syndrome (ARDS), a devastating illness characterized by severe hypoxemia and diffuse alveolar damage (1). ALI is triggered by a set of pulmonary and extrapulmonary etiologies (1, 2). Inflammation mediated by innate immunity plays an important role in the pathophysiology of ALI (3). Despite advances in organ support, wider availability of critical care services and improvements in the treatment of critical care syndromes, the impact of ARDS on patient-important outcomes remains high (4). Hence, the therapeutic treatment targeting the overwhelming inflammation in ALI remains urgent.

NLRP3 is the best characterized and most widely implicated inflammasome in innate immunity of lung inflammatory diseases (5). We previously demonstrated that NLRP3 activation is involved in the pathogenesis of ALI (6, 7). However, factors triggering the activation of the NLRP3 inflammasome in ALI are not fully understood.

IL-1β plays a potent, pyrogenic, pro-inflammatory role in initiating acute inflammatory response to infectious stimuli. These studies were primarily performed in macrophages (5). Type II alveolar epithelial cell (AEC II) is responsible for surfactant production and also serves as epithelial progenitors (8). Studies revealed that it also regulates several aspects of immune response (9, 10). Interestingly, it has been reported that IL-1β production appeared to predominantly originate from AEC II during lung ischemia-reperfusion inflammation (11). However, signals activating NLRP3 inflammasome in AEC II remains unknown.

Calcium (Ca^2+^), an important intracellular second messenger, is responsible for the control of numerous cellular processes (12). It has been identified that Ca^2+^ influx participates in NLRP3 inflammasome activation (13, 14). Ca^2+^ binding to calmodulin (CaM) induces conformational changes of CaM, leading to increased affinity of the Ca^2+^-CaM complex for its targets, CaMK (Ca^2+^/Calmodulin-activated Protein Kinases) (15). CaMK belongs to the serine/threonine kinase family that regulates gene expression by activating transcription factors including CREB (cAMP response-element binding protein) and CREM (cAMP response element modulator) (15, 16). Among the CaMK family, CaMK1 and CaMK2 are expressed ubiquitously, while CaMK4 is found predominately in cells of the immune and nervous systems (17). CaMK4 is important for immune responses (18), but their roles in the pathogenesis of ALI are not known. In this study, we found that AEC II expressed high level of CaMK4, which contributed to the activation of NLRP3 inflammasome in AEC II cell and promoted acute lung injury.

## Methods and Materials

### Mice

Male C57BL/6 mice (6-8 weeks old) were purchased from Beijing Vital River Laboratory Animal Technology Co., Ltd. (Beijing, China) and housed under a specific pathogen-free condition in the Experimental Animal Center of Sun Yat-sen University, Guangzhou, China. The mice were cared and used in accordance with the National Institutes of Health Guide for Care and Use of Animals. All procedures were approved by the Ethics Committee of Sun Yat-sen University.

### LPS-Induced ALI Model

ALI mouse model was established as described previously (6). Mice were injected with ketamine (80mg/kg) and xylazine (15mg/kg) intraperitoneally for anesthesia. Lipopolysaccharide (LPS, 6mg/kg, Sigma-Aldrich, USA) was delivered to the lungs of the mice *via* a 20-gauge angiocath catheter. The sham operated mice were given equal volume of PBS as control.

Mice were treated with KN-93 (10mg/kg, Cell Signing Technology, USA), a selective CaMK4 inhibitor or vehicle intraperitoneally. First injection was performed 2h before LPS administration. Mice were then treated with KN-93 or vehicle every 24 h for 48 h. Mice were euthanized 48 h after LPS administration. The bronchoalveolar lavage fluid (BALF) was then collected and stored for cell counting, total protein analysis, ELISA (enzyme linked immunosorbent assay) and histology. Lung tissues were collected for histology, immunohistochemistry, immunofluorescence, ELISA and Western blot analysis.

### Bronchoalveolar Lavage

BAL was performed as previously described (7). Mice were euthanized and the thoracic cavity was opened. The trachea was exposed and a small semi-excision was made. An 18G sterile needle with blunt end was inserted into the trachea through the excision. 2ml cold PBS was injected and a total volume of 2.4 ml BALF was collected from each mouse. Cell counts in the BALF were determined on a grid hemocytometer. Protein level in the BLAF was measured using a BCA Protein Assay Kit (Thermo Fisher Scientific, USA) according to the manufacturers’ instructions. BALF was collected from patients with ALI in the Department of Respiratory and Critical Care Medicine, the First Affiliated Hospital, Sun Yat-Sen University with approval of the Institutional Review Board.

### Immunohistochemistry

AEC II cells in the lungs were identified by the expression of pro-surfactant Protein C (pro-SPC) using immunohistochemical staining. Activation of CaMK4 in AEC II was detected using double immunohistochemical staining of pro-SPC (Abcam, UK, ab90716, dilution 1:200) and pCaMK4 (Thr196/200, Abcam, UK, ab59424, dilution 1:100). The staining of pCaMK4 in the lung tissue was visualized with DAB Detection Kit (brown chromogen, Gene Tech, Shanghai). The sequential pro-SPC/pCaMK4 double staining procedure was performed as we previously described (19). Briefly after pro-SPC staining with AEC Detection Kit (Abcam, UK) to produce a red color, the sections were heated to 100°C in microwave for 10 minutes to denature any bound antibodies. The sections were then stained with anti–pCaMK4 and visualized with NBT/BCIP Detection Kit (Abcam, UK) to produce a blue color.

### Cell Culture

Human AEC II line (A549) was purchased from the American Type Culture Collection (ATCC, USA). Cells were cultured in DMEM (Life Technologies, USA) supplemented with 10% FBS (Gibco, USA), 100 U/ml penicillin and 100 mg/ml streptomycin at 37°C, 5% CO_2_. To stimulate the activation of NLRP3 inflammasome in A549 cells, cells were primed with 10 μg/ml LPS for 8h. Cells were then stimulated with ATP (5 mM) for 1 h. CaMK4 inhibitor KN-93 was included in some of the experiments.

### Histology and Immunofluorescence

Lungs were fixed in 10% neutral formalin for 24 h. Lung tissues were embedded in paraffin and sectioned (2 μm), followed by hematoxylin and eosin (HE) staining. The slide from BALF of mice was stained by Wright-Giemsa staining. For tissue immunofluorescence staining, lung tissues were snap frozen in OCT and sectioned. Tissue slides and cytospin slide of BALF from patients with ALI were fixed in cold acetone and permeabilized in 0.01% Triton X-100. For A549 cell staining, cells were fixed in 4% paraformaldehyde and permeabilized in 0.01% Triton X-100. Slides were then stained with antibodies against pro-SPC (dilution 1:200) and FAM-FLICA Caspase-1 assay kit (ImmunoChemistry Technologies, USA) was used to detect activated Caspase-1. Slides with primary antibodies were incubated overnight at 4°C and then labeled with secondary anti-rabbit antibody conjugated with Alexa Fluor A555 (Thermo Fisher Scientific, USA, dilution 1:500) at room temperature for 60 min. Slides were then mounted with mounting media with 4′,6-diamidino-2-phenylindole (DAPI) and visualized using a fluorescence microscope LSM 800 (Zeiss, Jena, Germany).

### Western Blot

Proteins from lung tissues were extracted and analyzed by Western blotting. Total proteins were extracted with cell lysis buffer (Cell Signaling Technology, USA) according to the manufacturer’s instructions. Proteins were separated by electrophoresis and transferred to a polyvinylidene difluoride membrane (Millipore, Germany). Membranes were then blocked with 5% bovine serum albumin and incubated with primary antibodies against NLRP3 (AdipoGen, USA, clone: Cryo-2, dilution: 1:1000), Caspase-1 p20 (AdipoGen, USA. clone: Casper-1, Dilution: 1:1000), pCaMK4 (dilution 1:1000) at 4°C overnight. GAPDH (Cell Signaling Technology, USA, clone: 14C10, dilution 1:2000) was used as internal control. Membranes were washed and incubated with horseradish peroxidase-conjugated anti-mouse or anti-rabbit IgG (both from Cell Signaling Technology, USA, dilution: 1:2000). Signals were detected with enhanced chemiluminescence analysis kit (Cell Signaling Technology, USA).

### Transfection

Small interfering RNA duplexes targeting CaMK4 (si-CaMK4, Ribobio, Guangzhou, China) were synthesized for cell treatment. Cells were transfected with 100 nM siRNA or scramble siRNA together with Lipofectamine RNAiMAX (Invitrogen, USA) cultured in complete medium for 48 h before stimulation.

### Enzyme-Linked Immunosorbent Assay

IL-1β and IL-18 in lung tissues, BALF and serum were measured using ELISA kits for mouse and human IL-1β and IL-18 (R&D Systems, Minneapolis, USA) following the manufacturers’ instructions.

### Statistical Analysis

Data are presented as means ± standard error of mean (SEM). Statistical analyses were conducted using GraphPad Prism 8.0. The differences were assessed by Student’s *t* test, or one-way ANOVA as appropriate followed by adjustment of multiple comparisons. Two-tailed p < 0.05 was considered statistically significant.

## Results

### Activation of CaMK4 and NLRP3 Inflammasome in ALI

**Figure 1:**
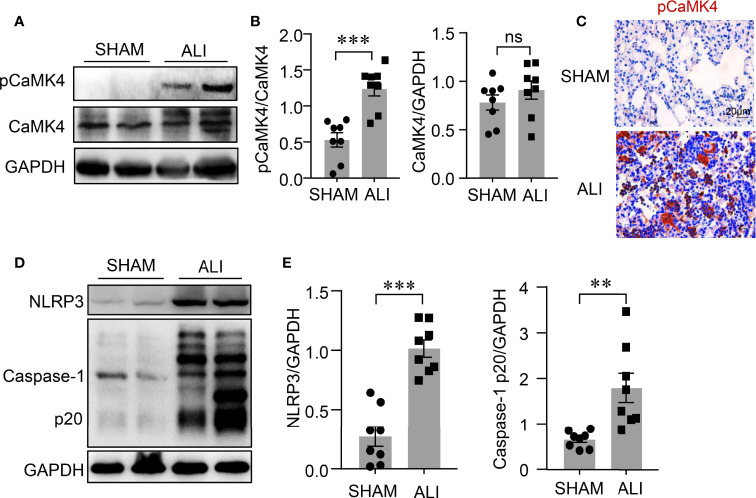
Activation of CaMK4 and NLRP3 inflammasome in LPS-induced ALI. LPS (6mg/kg) was delivered to the lungs of the C57BL/6 mice and sham operated mice were given equal amount of PBS as control. Lung tissues were collected 2 days after mice treated with PBS (SHAM) or LPS (ALI). **(A)** Representative bands of immunoblotting showed p-CaMK4 and CaMK4 expression in lung tissues from PBS or LPS treated mice. **(B)** Relative expression of p-CaMK4 to CaMK4 was summarized from 8 mice. **(C)** Immunohistochemistry staining of pCaMK4 in lung tissues from PBS or LPS treated mice. Representative images were shown. Scale bar: 20μm. **(D)** NLRP3, Caspase-1p20 and GAPDH in the lung tissues from mice treated with PBS or LPS were measured by Western blot. Representative bands were shown. **(E)** Relative expressions of NLRP3 and Caspase-1p20 to GAPDH were summarized. All data are mean ± SEM. **p < 0.01, ***p < 0.001. ns, not significant.

The NLRP3 inflammasome is involved in the pathogenesis of ALI, but the underlying mechanism is unclear. CaMK4 plays an important role in immune responses (18). A LPS-induced ALI mouse model was used to examine the correlation between CaMK4 and the activation of NLRP3 inflammasome. The phosphorylation of CaMK4 was significantly increased in the lung tissue of LPS-induced ALI mice compared to control mice as measured by Western blot (**Figures 1A, B**). Immunohistochemistry showed enhanced staining of pCaMK4 in the lung tissues of LPS-treated mice compared to PBS-treated mice (**Figure 1C**). Interestingly, NLRP3 inflammasome was activated in parallel with the activation of CaMK4 in the lung tissue of LPS-induced ALI. The level of cleaved Caspase-1 p20, an indicator for the activation NLRP3 inflammasome, was significantly upregulated in the lung tissue of ALI mice as measured by Western blot (**Figures 1D, E**). These data suggest that CaMK4 might be involved with the regulation of NLRP3 inflammasome activation in ALI.

### CaMK4 Regulated the Activation of NLRP3 Inflammasome in Acute Lung Injury

**Figure 2:**
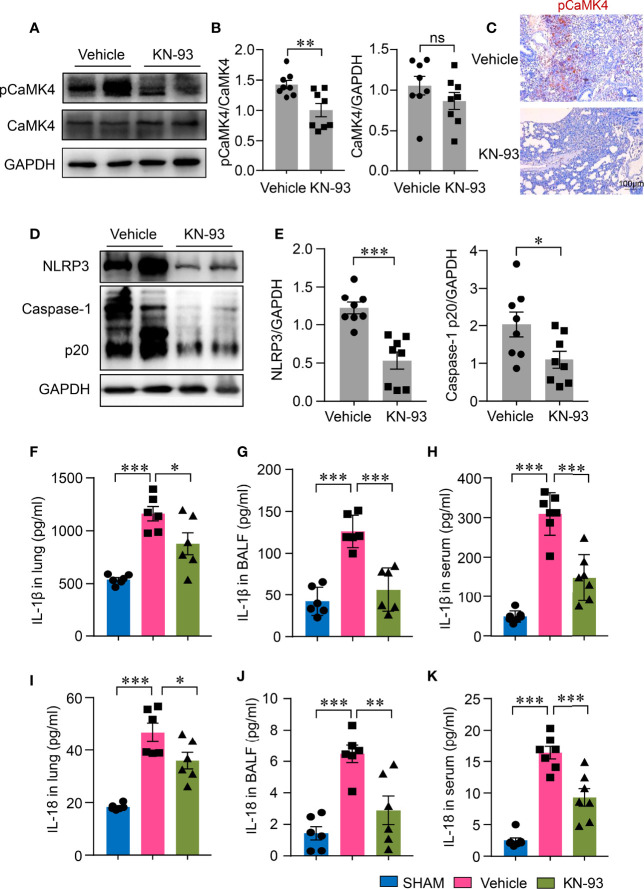
KN-93 inhibited the activation of NLRP3 inflammasome in ALI. ALI mice were induced as described in Fig. 1 and treated with KN-93 (10mg/kg) or vehicle. **(A, B)** Expressions of pCaMK4 and CaMK4 in the lung tissues from KN-93 or vehicle treated mice were measured by Western blot. Representative bands were shown. Relative expression of pCaMK4 was quantified. **(C)** Immunohistochemical staining of pCaMK4 in lung tissues from KN-93 or vehicle treated mice. Representative images were shown. Scale bar: 100μm. **(D, E)** NLRP3, Caspase-1 p20 and GAPDH in the lung from KN-93 or vehicle treated mice were measured by Western blot. Representative bands were shown and relative expression of NLRP3, Caspase-1p20 to GAPDH were summarized. **(F–K)** IL-1β and IL-18 in lung tissues, bronchoalveolar lavage fluid (BALF) and sera from KN-93 or vehicle treated mice were measured by ELISA. All data are mean± SEM. *p < 0.05, **p < 0.01, ***p < 0.0001. ns, not significant.

To investigate whether CaMK4 plays a role in NLRP3 inflammasome activation during LPS-induced ALI, ALI mice were treated with a CaMK4 specific inhibitor, KN-93. KN-93 treatment inhibited the phosphorylation of CaMK4 in the lung tissue of ALI as measured by Western blot (**Figures 2A, B**). Immunohistochemistry further confirmed the reduction of pCaMK4 in the lung tissues of KN-93 treated mice as compared to PBS-treated mice (**Figure 2C**). Accordingly, NLRP3 inflammasome activation in the lung tissue was also significantly suppressed by CaMK4 inhibition. NLRP3 expression and the cleavage of Caspase-1 into p20 were significantly reduced by KN-93 (**Figures 2D, E**). These data suggest that CaMK4 regulate the activation of NLRP3 inflammasome in the lung tissue of LPS-induced ALI.

### CaMK4 Inhibition Decreased NLRP3 Inflammasome-Related Cytokine Production Locally and Systemically

The activation of NLRP3 inflammasome leads to the maturation of IL-1β and IL-18, both of which have been implicated in the pathology of ALI. To study whether CaMK4 inhibition affects the production of IL-1β and IL-18 in ALI, we measured IL-1β and IL-18 in the lungs of ALI. IL-1β and IL-18 levels both in the BALF and in the lung tissues were both significantly increased in ALI mice when compared to normal mice. The levels of IL-1β and IL-18 in the BALF and in the lung tissues were significantly reduced by KN-93 (**Figures 2F, G, I, J**). Moreover, CaMK4 inhibition ameliorated systemic inflammation in that serum concentrations of IL-1β and IL-18 were also lower in KN-93 treated mice (**Figures 2H, K**). These data demonstrate that CaMK4 plays a role in NLRP3 inflammasome activation in ALI.

### Activation of CaMK4 and NLRP3 Inflammasome in Type II Lung Alveolar Epithelial Cells in ALI.

**Figure 3:**
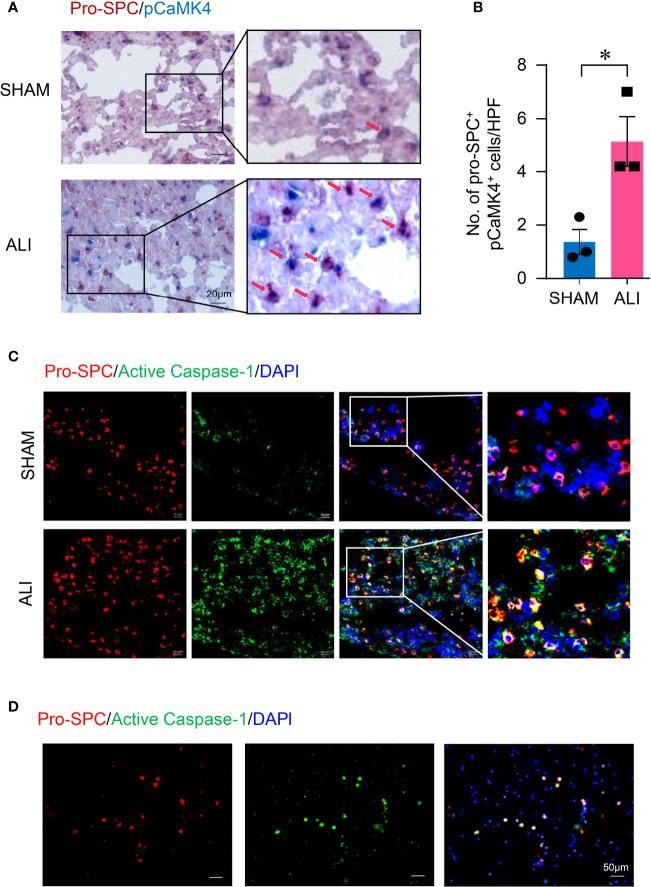
Activation of CaMK4 and NLRP3 inflammasome in type II lung alveolar epithelial cells in ALI. **(A, B)** Lungs were collected from ALI or normal control mice and sectioned. pro-SPC (red) and pCaMK4 (blue) expression in the lung were identified by immunohistochemistry. Representative images were shown and numbers of pro-SPC^+^pCaMK4^+^ cells (arrows) in the lung tissues per high power field (HPF) were summarized from 3 samples. Scale bar: 20μm. **(C)** Slices from lungs of ALI or control mice were stained with Alexa Fluor A555-conjugated anti-pro-SPC (red) and FAM-FLICA (green). Representative images were shown. Scale bar: 20μm. **(D)** Bronchoalveolar lavage fluid (BALF) was collected from patients with ARDS and cells were isolated by centrifugation. Cells were stained with Alexa Fluor A555-conjugated pro-SPC (red) and FAM-FLICA (green). Representative images were shown. Scale bar: 50μm. All data are mean± SEM. *p < 0.05.

It has been reported that AEC II represented the major source of IL-1β and promoted inflammation in the lung during ischemia–reperfusion (11). To investigate whether AEC II contribute to the increased activation of CaMK4 and NLRP3 inflammasome in LPS-induced ALI, lung tissue slides from ALI or control mice were first measured for pCaMK4 expression by immunohistochemistry. Mouse AEC II were labeled with pro-SPC. AEC II from LPS-induced ALI were strong positive for pCaMK4 (**Figures 3A, B**), indicating the activation of CaMK4 in AEC II cells during ALI. Meanwhile, tissue slides from ALI or control mice were stained with antibodies against pro-SPC and FLICA Caspase-1 probe, which binds to activated Caspase-1 irreversibly. AEC II from LPS-induced ALI were also strongly positive for activated Caspase-1 as determined by immunofluorescence. In contrast, the activated Caspase-1 was hardly detected in the AEC II from control mice (**Figure 3C**). The activation of NLRP3 inflammasome in AEC II was further confirmed in ALI patient-derived samples. BALF was collected from patients with ARDS. Immunofluorescence staining demonstrated that AEC II from patients with ARDS expressed high level of activated Caspase-1 (**Figure 3D**). Together, these data indicate that NLRP3 inflammasome was activated in AEC II during ALI.

### CaMK4-Mediated NLRP3 Inflammasome Activation in Type II Alveolar Epithelial Cells

**Figure 4:**
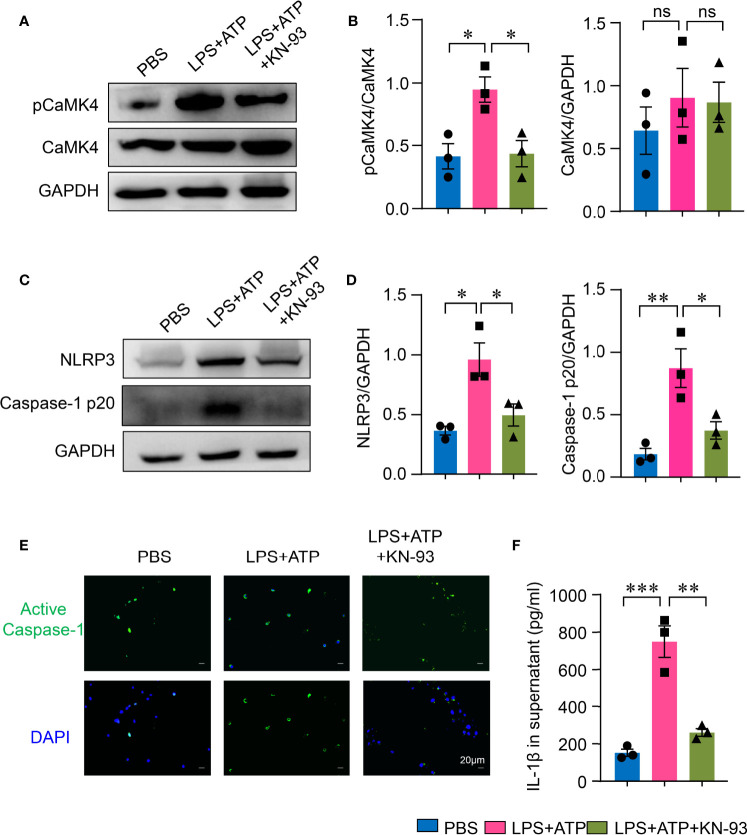
KN-93 inhibited NLRP3 inflammasome activation in type II lung alveolar epithelial cells. A549 cells were stimulated with LPS + ATP or PBS. KN-93 was included in some of the experiments. **(A, B)** The expression of pCaMK4, CaMK4 and GAPDH was measured by Western blot. Representative bands were shown and relative expression was summarized from 3 independent experiments. **(C, D)** NLRP3, Caspase-1 p20 and GAPDH expression was measured by Western blot. Representative bands and relative expression were shown. **(E)** Immunofluorescence showed the activated Caspase-1 (green) in A549 cells. Representative images were shown. Scale bar: 20μm. **(F)** IL-1β in supernatants from A549 cells was determined by ELISA. All data are mean± SEM. *p < 0.05, **p < 0.01, ***p < 0.001. ns, not significant.

To further investigate the roles of CaMK4 in NLRP3 inflammasome activation in AEC II, AEC II were first treated with CaMK4 inhibitor KN-93. KN-93 effectively suppressed the phosphorylation of CaMK4 in AEC II cells (**Figures 4A, B**), which led to the inhibition of NLRP3 inflammasome activation. NLRP3 and Caspase-1p20 expression in AEC II was significantly inhibited by KN-93 as measured by Western blot (**Figures 4C, D**). Furthermore, activated Caspase-1 in AEC II was reduced by KN-93 as measured by immunofluorescence (**Figure 4E**). As a result, the inhibition of NLRP3 inflammasome by KN-93 led to decreased production of IL-1β (**Figure 4F**).

**Figure 5:**
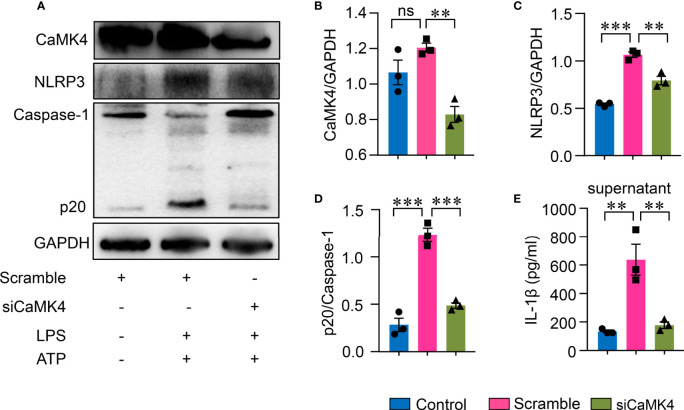
Knockdown of CaMK4 inhibited NLRP3 inflammasome activation in type II lung alveolar epithelial cells. CaMK4 expression in A549 cells were knocked down using CaMK4 specific siRNA and stimulated with LPS+ATP or PBS. **(A–D)** CaMK4, NLRP3, Caspase-1p20 and GAPDH expression was measured by Western blot. Representative bands were shown and relative expression summarized from 3 independent experiments. **(E)** IL-1β in supernatants from A549 cells was determined by ELISA. All data are mean± SEM. ns, not significant. **p < 0.01, ***p < 0.001.

To further confirm the role of CaMK4 in NLRP3 inflammasome activation in AEC II, CaMK4 was knocked down using CaMK4 specific siRNA. Knockdown efficiency was confirmed by Western blot (**Figures 5A, B**). NLRP3 inflammasome activation was suppressed when CaMK4 was knocked down by siRNA. NLRP3 and Caspase-1p20 expression was significantly decreased by CaMK4 siRNA (**Figures 5A, C, D**). IL-1β released to the supernatant was also significantly reduced by knocking down of CaMK4 (**Figure 5E**). These data demonstrate that CaMK4 controls the activation of NLRP3 inflammasome in AEC II cells.

### CaMK4 Inhibition Decreased NLRP3 Inflammasome Activation in Type II Alveolar Epithelial Cells and Ameliorated Acute Lung Injury

**Figure 6:**
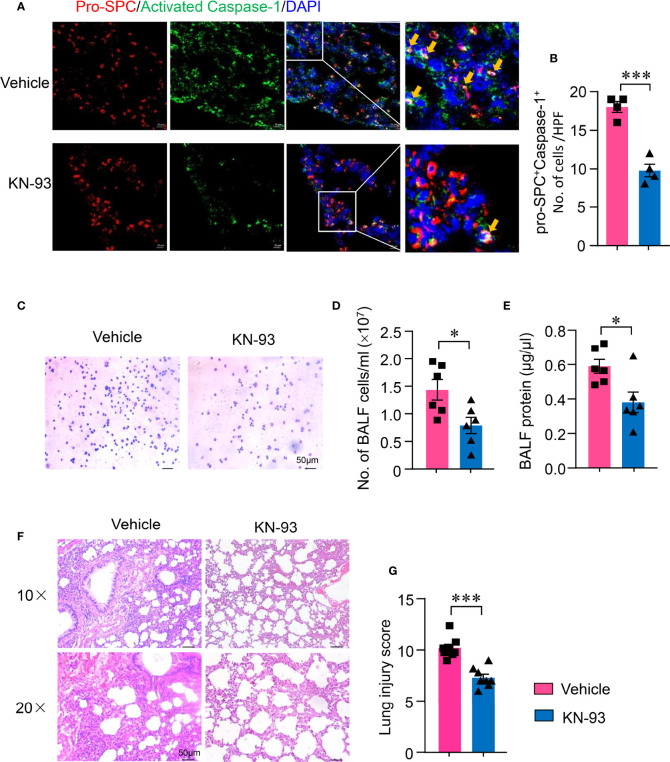
KN-93 inhibited NLRP3 activation in type II alveolar epithelial cells and ameliorated acute lung injury. Sections from lung tissues of ALI mice treated with KN-93 or vehicle were stained with Alexa Fluor A555-conjugated anti-pro-SPC and FAM-FLICA. **(A)** Representative images showing pro-SPC (red) and activated Caspase-1 (green) expression in lung tissues. Scale bar: 20μm. **(B)** The number of pro-SPC^+^FLICA^+^ cells per high power field in the lung tissues were summarized from 4 mice. **(C)** Cytospin cells were collected from bronchoalveolar lavage fluid (BALF) and Wright-Gimsa staining was performed. Representative images were shown. Scale bar: 50μm. **(D)** Total cell counts in BALF from KN-93 or vehicle treated mice. **(E)** Protein concentration in BALF was measured using a BCA assay. **(F, G)** HE staining of lung sections from KN-93 or vehicle treatment ALI mice. Representative images. Scale bar: 50μm. Lung injury score was calculated and summarized from 8 mice. All data are mean± SEM. *p < 0.05, ***p < 0.001.

To investigate whether CaMK4-controlled NLRP3 inflammasome activation in AEC II contributes to the development of ALI, ALI mice were treated with KN-93. NLRP3 inflammasome activation in AEC II of ALI was determined by immunofluorescence. KN-93 significantly suppressed NLRP3 inflammasome activation in AEC II. Activated Caspease-1 was reduced in AEC II of mice treated with KN-93 (**Figures 6A, B**). In addition, inflammatory infiltrates to the alveolar was measured by BAL-derived cell staining. The suppression of CaMK4-NLRP3 inflammasome pathway by KN-93 in AEC II resulted in decreased infiltration of mononuclear cells and granulocytes (**Figures 6C, D**). The protein exudate in the alveolar was also decreased by KN-93 (**Figure 6E**). Importantly, the suppression of CaMK4-NLRP3 inflammasome pathway in AEC II resulted in amelioration of lung injury. Inflammatory infiltrates and pathologic score were significantly reduced by CaMK4 inhibition as evaluated histologically (**Figures 6F, G**).

## Discussion

Overwhelming inflammation mediated by innate immunity plays a pivotal role in the pathogenesis of ALI. As an early mediator for inflammation, NLRP3 inflammasome is believed to exhibit pro-inflammatory activities in ALI (20, 21). Caspase-1 activation and the subsequent production of IL-1β were identified in both ALI animal models and patients (6, 22). Our previous studies revealed that inhibition P2X7 (6) or RIP3 (23), which acts as upstream of NLRP3 inflammasome, suppressed inflammatory cell infiltration and cytokine production in LPS-induced ALI mice. However, the precise mechanism of NLRP3 inflammasome activation has not been fully elucidated yet. Calcium (Ca^2+^) is considered as a pervasive intracellular second messenger that initiates signaling cascades and operating essential biologic processes, such as exocytosis, metabolism, cell fertilization, proliferation and contraction (24). During sepsis, disturbed cellular calcium homeostasis is assumed to mediate the aberrant inflammation underlying organ dysfunction and death (25). Binding of calcium to CAM leads to activation of CAMK which is thought to be a novel route for inflammation initiation (17). In this study, we firstly demonstrated that the activation of CaMK4 was in parallel with the inflammatory lung injuries in LPS-challenged mice. CaMK4 is involved in NLRP3 inflammasome activation during ALI.

CaMK4, as a downstream multifunctional kinase of Ca^2+^ signaling cascades, is considered as a necessary component for function of calcium in immune response and inflammation and regulates various cellular events through phosphorylation of transcription factors (17, 26). In cardiotoxin-induced acute skeletal muscle inflammatory response, CaMK4 has been implicated in infiltrated mononuclear cells and regenerated myofibers (27). This is also reported in primary macrophage isolated from CaMK4^-/-^ mice, in which serine phosphorylation of proinflammatory cytokines HMGB1 in response to LPS was inhibited (28). These findings suggest CaMK4 might elicit inflammation during ALI. In this study, we found pCaMK4 expression was significantly enhanced in the lung tissues of ALI mice, paralleled with alveolar damage and inflammatory cytokine production. Inhibition of CaMK4 alleviated neutrophil accumulation and production of proinflammatory cytokines, resulting in amelioration of lung damage, all of which indicate that the CaMK4/NLRP3 pathway is implicated in the pathogenesis of ALI. The precise molecular mechanisms underlying the CaMK4 enrolled inflammation awaits to be established.

Several molecular mechanisms, including pore formation and potassium (K^+^) efflux, lysosomal destabilization and rupture, and mitochondrial reactive oxygen species (ROS) generation, have been suggested for NLRP3 inflammasome activation to induce Caspase-1 activation and IL-1β maturation (29). Recent studies indicate that elevated levels of calcium functioned as a signal for the maturation of proinflammatory cytokine IL-1β by stimulating the assembly of multiprotein inflammasomes (30). We found that NLRP3 inflammasome was activated in the lungs of ALI mice accompanied with enhanced expression of pCaMK4. CaMK4 specific inhibitor KN-93 suppressed NLRP3 inflammasome activation, indicating the activation of CaMK4 is engaged in the NLRP3 inflammasome activation.

Functions and regulations of inflammasomes have been studied mainly on macrophages or DCs (5). Recent studies revealed that AEC II, also acting as an innate immunocyte, could regulate the activation of NLRP3 inflammasomes and IL-1β production in lung inflammation (11, 31, 32). In our study, activated Caspase-1 was found to be colocalized and overlap with pro-SPC, a marker for AEC II, in the lung tissues of ALI mice as well as in cells of alveolar lavage fluids from ARDS patients, indicating that AEC II produces activated Caspase-1 in the injured lungs. Proinflammatory effects of CaMK4 have been demonstrated in circulating lymphocytes (17). CaMK4 is also reported to be expressed in intrinsic cells of various tissues, such as podocyte, myoblasts, amygdala, hippocampus cells (27, 33–35), whether AEC II expresses CaMK4 has not been reported. In this study, we found that AEC II expressed CaMK4 and enhanced activation of CaMK4 was noted in the lung tissues of ALI mice. To further explore whether CaMK4 promotes NLRP3 inflammasome activation in AEC II and contributes to the progression of ALI, KN-93 was used to treat the ALI mice *in vivo* and the AEC II cell line *in vitro*. Expression of activated Caspase-1 and production of proinflammatory cytokines were significantly decreased after KN-93 treatment. The similar result was achieved by silencing CaMK4 expression using CaMK4 siRNA. These data demonstrated that CaMK4 promotes NLRP3 activation in AEC II during ALI.

In summary, our data reveal that CaMK4 participates in NLRP3 inflammasome activation in AEC II and promotes lung injury in LPS-induced ALI mice. Inhibition of CaMK4 by KN-93 effectively ameliorated ALI by inhibiting NLRP3 inflammasome activation. Targeting CaMK4-NLRP3 signaling pathway may be used a potential therapeutic strategy for human ALI.

## Data Availability Statement

The original contributions presented in the study are included in the article/supplementary material. Further inquiries can be directed to the corresponding author.

## Ethics Statement

The studies involving human participants were reviewed and approved by the Ethics Committee of the First Affiliated Hospital, Sun Yat-sen University. Written informed consent to participate in this study was provided by the participants’ legal guardian/next of kin. The animal study was reviewed and approved by the Ethics Committee of the Laboratory Animal Center of Sun Yat-sen University.

## Author Contributions

TZ, ML, SZ, MZ, HL, HWu, XM, HWang, CG and YH performed the experiments. YH designed the study. TZ, ML and YH analyzed the data. TZ, ML, HZ, NY and YH wrote the paper. All authors read and approved the manuscript.

## Funding

This project was supported by grants from Natural Science Foundation of Guangdong Province (2021A1515012072) and by the National Natural Science Foundation of China (81971519 and 82171770).

## Conflict of Interest

The authors declare that the research was conducted in the absence of any commercial or financial relationships that could be construed as a potential conflict of interest.

## Publisher’s Note

All claims expressed in this article are solely those of the authors and do not necessarily represent those of their affiliated organizations, or those of the publisher, the editors and the reviewers. Any product that may be evaluated in this article, or claim that may be made by its manufacturer, is not guaranteed or endorsed by the publisher.
